# Supplementary material for: Autophagy acts as a brake on obesity-related fibrosis by controlling purine nucleoside signalling
Source: Nat Commun. 2025 Oct 17;16:9220. doi: 10.1038/s41467-025-64266-5 (PMC12534472; doi:10.1038/s41467-025-64266-5)
Supplement: Supplementary file 1 — Supplementary Information [file 41467_2025_64266_MOESM1_ESM.pdf]

SUPPLEMENTARY INFORMATION

Autophagy acts as a brake on obesity-related fibrosis by controlling purine nucleoside signalling

Klara Piletic[1], *Amir H. Kayvanjoo*[2,3,#], *Felix Clemens Richter*[1,#], *Mariana Borsa*[1,$], *Ana V. Lechuga-Vieco*[1,$], *Oliver Popp*[2], *Sacha Grenet*[1,4], *Jacky Ka Long Ko*[5], *Lin Luo*[1,6], *Kristina Zec*[1], *Maria Kyriazi*[7], *Harriet K. Haysom*[1], *Lada Koneva*[1], *Stephen Sansom*[1], *Philipp Mertins*[2], *Fiona Powrie*[1], *Ghada Alsaleh*[7,Φ], *Anna Katharina Simon*[1,2,Φ*]

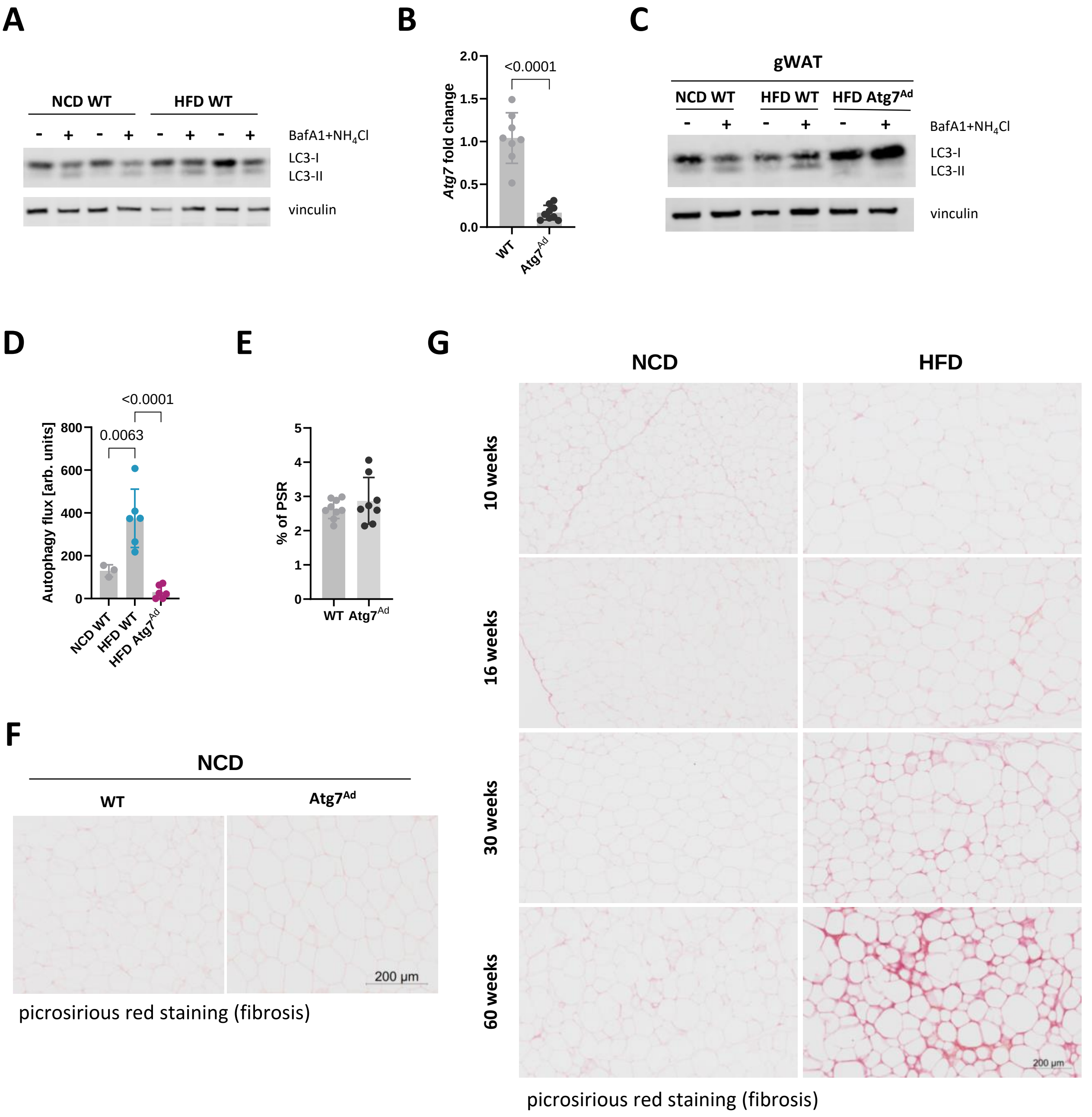

Supplementary Figure 1: Assessment of autophagy flux and fibrosis in obesity and validation of *Atg7^Ad^* mouse model.

A) A representative western blot analysis of LC3-I, LC3-II, and vinculin in gWAT to measure autophagy flux. WT mice were fed a normal chow diet (NCD) or high fat diet (HFD) for 10, 30 and 60 weeks. An example western blot is shown for 30 weeks of altered diet.

B) Relative mRNA expression of *Atg7* in gWAT of WT and *Atg7*^{Ad} mice measured by qRT-PCR. n = 8 (WT) and 9 (*Atg7*^{Ad}) mice.

C) A representative western blot analysis of LC3-I, LC3-II, and vinculin in gWAT to measure autophagy flux. WT and $Atg7^{Ad}$ mice were fed a normal chow diet (NCD) or high fat diet (HFD) for 16 weeks.

D) WT and $Atg7^{Ad}$ mice were fed a normal chow diet (NCD) or high fat diet (HFD) for 16 weeks before autophagy flux in gWAT was assessed as explained in Materials and Methods. Western blot analysis of autophagy flux (as in B) was calculated as (LC3-II (Inh) – LC3-II (Veh)). n = 3 (NCD) and 6 (HFD) mice.

E) Quantification of picrosirius red positive area as a percentage of the total area of stained gWAT. n = 8 ($Atg7^{Ad}$) and 9 (WT) mice.

F) Picrosirius red staining (PSR), specifically staining collagen I and III, of gWAT depots harvested from NCD-fed WT and $Atg7^{Ad}$ mice after 16 weeks of feeding. Scale bar, 200 µm.

G) PSR staining of gWAT depots harvested from NCD and HFD-fed WT mice after 10, 30, and 60 weeks of feeding. Scale bar, 200 µm.

Data are presented as mean ± SD. Dots represent individual biological replicates. Data are representative (A, C-D, F-G) or merged from 3 independent experiments (B, E). Statistical analysis by unpaired t-test (B) and one-way ANOVA with Tukey multiple comparisons test (D).

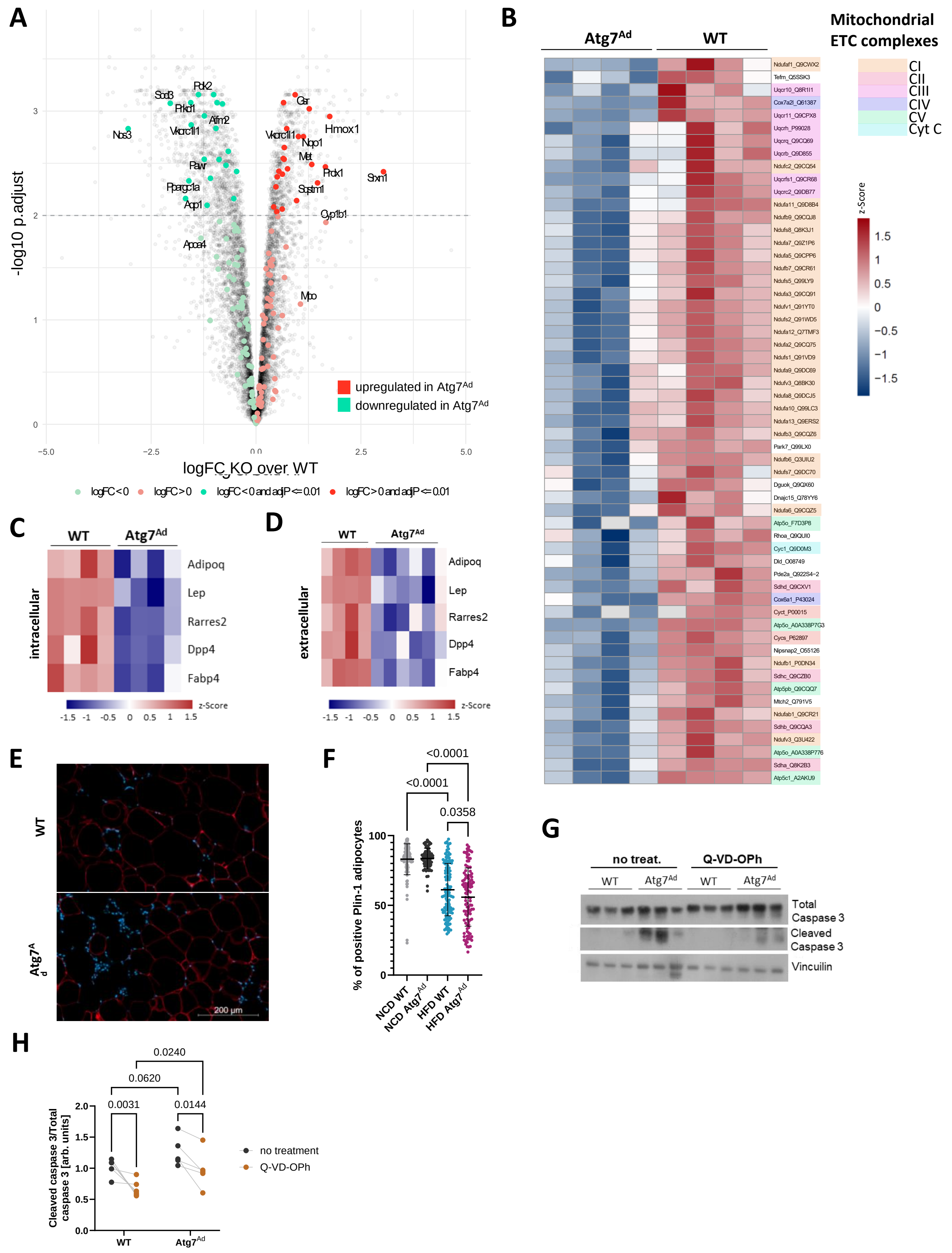

Supplementary Figure 2: Loss of autophagy induces a stress response and profoundly disturbs mitochondrial homeostasis, accompanied by increased cell death.

A) Vulcano plot of cellular response to oxidative stress (GO:0034599). n = 4 mice.

B) Heatmap of OXPHOS biological process (GO:0006119). Proteins belonging to different mitochondrial complexes are colour-coded. Values were scaled by row (protein) using z-score. n = 4 mice.

C-D) Heatmap showing intracellular (C) and extracellular (D) adipokine accumulation. Lep = leptin, Adipoq = adiponectin. Values were scaled by row (protein) using z-score. n = 4 mice.

E-F) Representative immunofluorescence staining of Perilipin-1 on gWAT sections from WT and $Atg7^{Ad}$ mice following HFD feeding for 16 weeks (E). Quantification of Perilipin-1 staining as a percentage of Perilipin-1 positive adipocytes compared to total area (F) from (E). Legend: blue = dapi, red = Perilipin-1. n = 7 (NCD) and 8 (HFD) mice, each section quantified across multiple areas of interest.

G-H) Cleaved caspase 3 was assessed by western blot analysis in gWAT explants cultured overnight *ex vivo* and treated with either DMSO or 20 µM Q-VD-OPh, a pan-caspase inhibitor. gWAT was isolated from WT and $Atg7^{Ad}$ mice fed with HFD for 16 weeks. G) Representative western blot analysis of total caspase 3, cleaved caspase 3, and vinculin in gWAT to measure apoptosis. H) Quantification of cleaved caspase 3 compared to total caspase 3 quantified from blots in (G). n = 5 ($Atg7^{Ad}$) and 6 (WT) mice.

Dots represent individual biological replicates. Data are representative (E, G) or merged from 2-3 independent experiments (F, H). Statistical analysis by Tukey's multiple comparisons test (F) or uncorrected Fisher's LSD test (H).

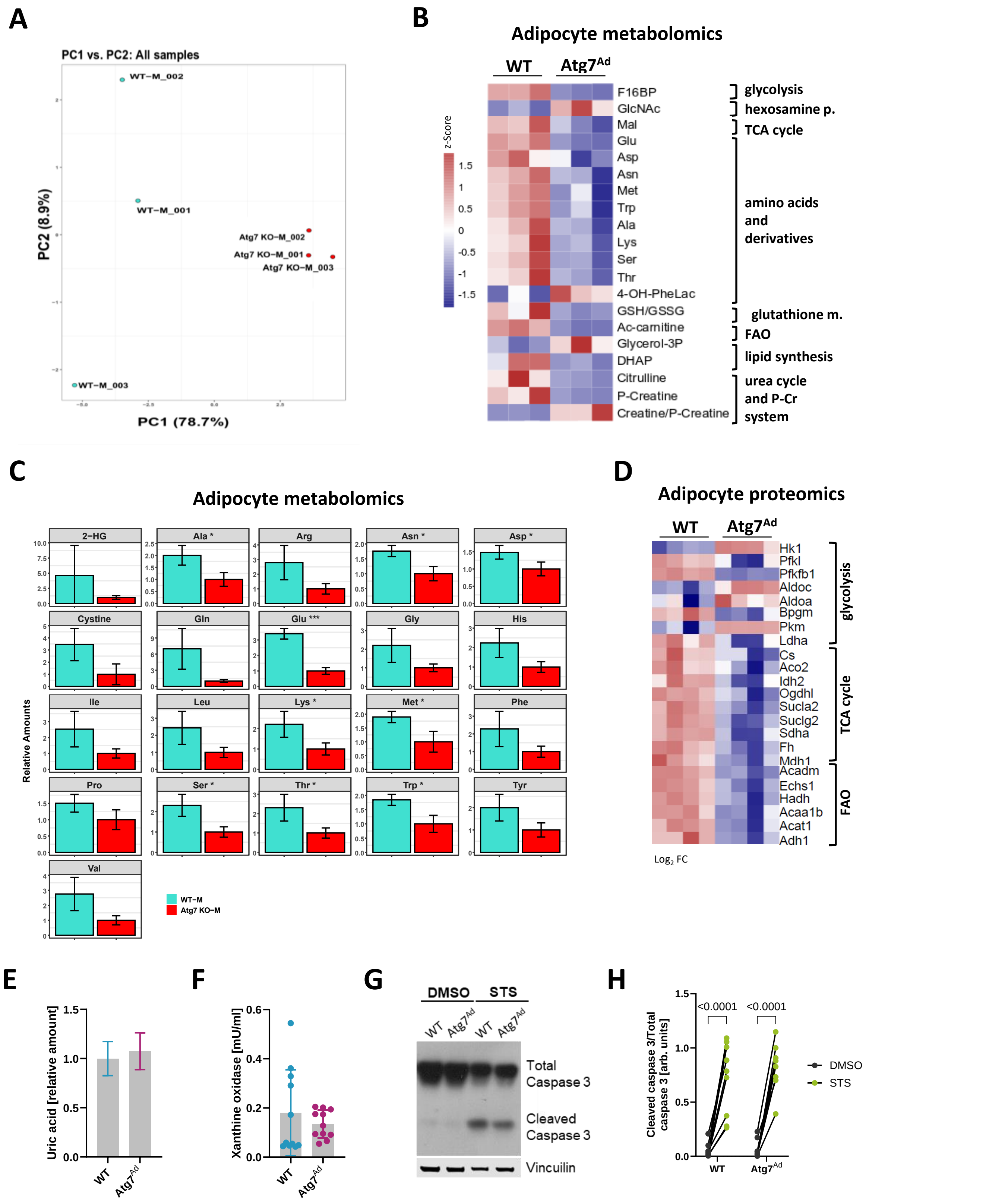

Supplementary Figure 3: Metabolomics analysis reveals a prominent role of autophagy in amino acid metabolism.

A) Principal component analysis (PCA) representation of metabolomics analysis. n = 3 mice per genotype, males only.

B) Z-score heatmap of significantly ($p < 0.05$) abundant metabolites in adipocytes. Metabolomics analysis was performed on adipocytes isolated from gWAT of WT and *Atg7*^{Ad} mice following HFD feeding for 16 weeks. n = 3 mice.

C) Relative abundance of amino acids measured with metabolomics analysis in adipocytes isolated from gWAT of WT and *Atg7*^{Ad} mice following HFD feeding for 16 weeks. n = 3 mice.

D) Log_2 fold change heatmap of significantly differentially abundant proteins between WT and *Atg7*^{Ad} involved in glycolysis, TCA cycle and FAO in adipocytes as measured by proteomics analysis. n = 4 mice.

E) Relative abundance of uric acid measured with metabolomics analysis in adipocytes isolated from gWAT of WT and *Atg7*^{Ad} mice following HFD feeding for 16 weeks. n = 3 mice.

F) Activity of xanthine oxidase (XO) after 2-hour incubation with WT and *Atg7*^{Ad} adipocyte lysates. n = 11 mice.

G-H) WT and *Atg7*^{Ad} mice were fed HFD for 16 weeks. gWAT explants were cultured over 24 hours *ex vivo* while treated with either DMSO or 10 µM staurosporine (STS), an apoptosis inducer. G) Representative western blot analysis of total caspase 3, cleaved caspase 3, and vinculin in gWAT. H) Cleaved caspase 3 to measure apoptosis was quantified from blots in (G). n = 8 (*Atg7*^{Ad}) and 9 (WT) mice.

All heatmap values were scaled by row (protein/metabolite) using z-score. Data are presented as mean ± SD. Dots represent individual biological replicates. Data are representative (G) or merged from 3 independent experiments (F, H). Statistical analysis by two-way ANOVA with Šídák multiple comparisons test (H).

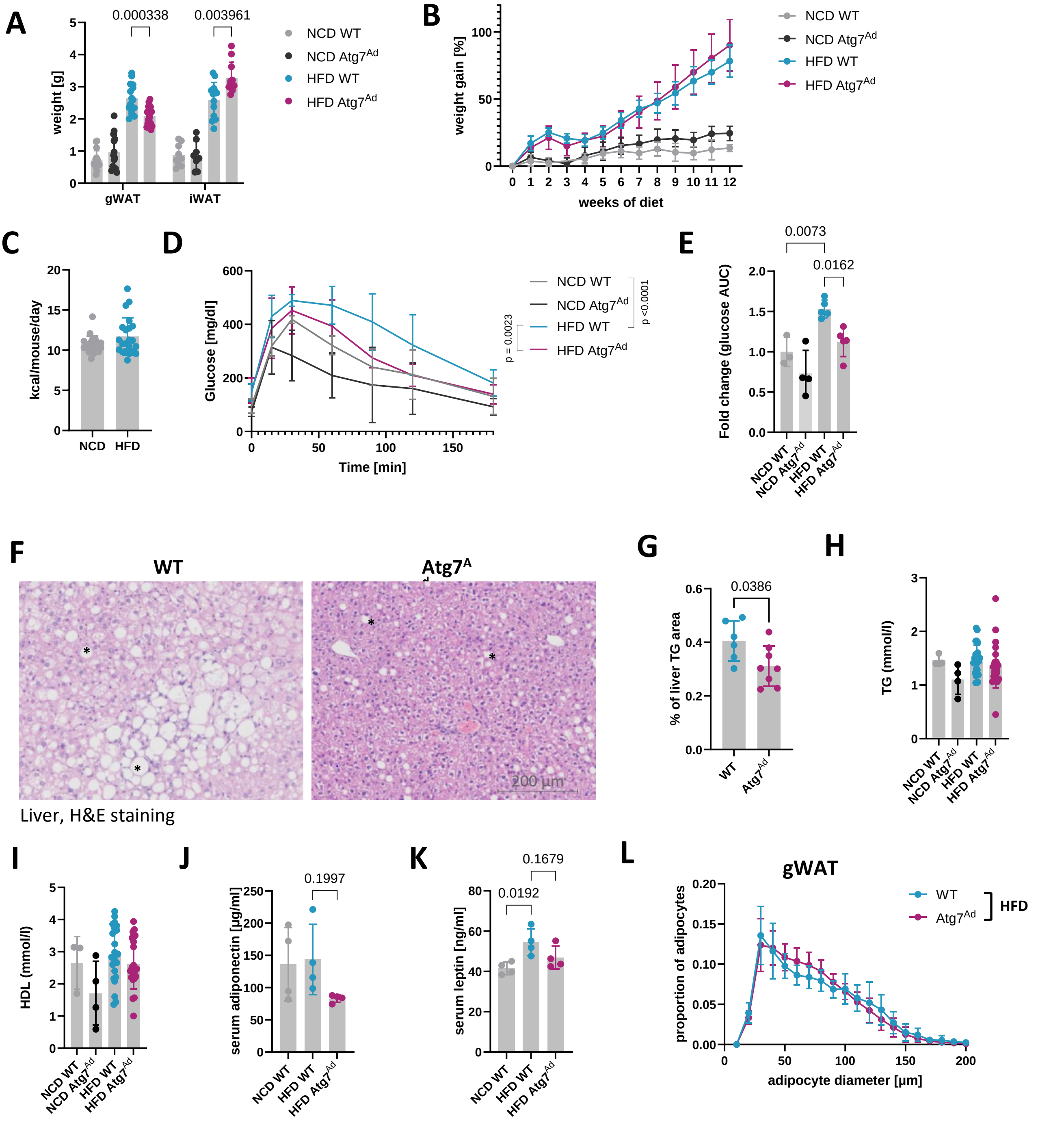

Supplementary Figure 4: The absence of adipocyte autophagy ameliorates metabolic syndrome in diet-induced obese mice.

WT and *Atg7^Ad^* mice were fed normal chow diet (NCD) or high fat diet (HFD) for 12-16 weeks prior to analysis.

A) Fat pad weight. n = 8 (iWAT NCD *Atg7^Ad^*), 9 (iWAT NCD WT), 11 (iWAT HFD *Atg7^Ad^*), 13 (iWAT HFD WT) and 15 (gWAT) mice.

B) Weight gain relative to starting weight over 12 weeks of NCD or HFD feeding. n = 4 mice.

C) Caloric food intake between NCD and HFD-fed mice. n = 22 mice.

D) Glucose tolerance test performed at 12 weeks of HFD feeding. n = 4 mice.

E) Area under the curve (AUC) for glucose tolerance test for (E).

F) H&E staining of liver harvested from HFD-fed WT and $Atg7^{Ad}$ mice after 16 weeks of feeding. Examples of fat deposition marked with an asterisk, scale bar, 200 µm.

G) Quantification of white (lipid) area from G. n = 6 (WT) and 8 ($Atg7^{Ad}$) mice.

H-K) Serum concentration of triglycerides (TG, H), high-density lipoprotein (HDL, I), adiponectin (J), and leptin (K). n = 3 (H – NCD WT, I – NCD WT), 4 (H – NCD $Atg7^{Ad}$, I – NCD $Atg7^{Ad}$, J, K), 22 (H – HFD $Atg7^{Ad}$, I – HFD $Atg7^{Ad}$) and 24 (H – HFT WT, I – HFD WT) mice.

L) Quantification of adipocyte number and diameter in gWAT from HFD-fed mice. n = 6 mice.

Data are presented as mean ± SD. Dots represent individual data points. Data are representative (B, D-F) or merged from 2-3 independent experiments (A, C, G-K, L). Statistical analysis by multiple unpaired t tests (A), two-way ANOVA with Tukey multi comparisons test (D, E), unpaired t-test (G), and one-way ANOVA (J-K).

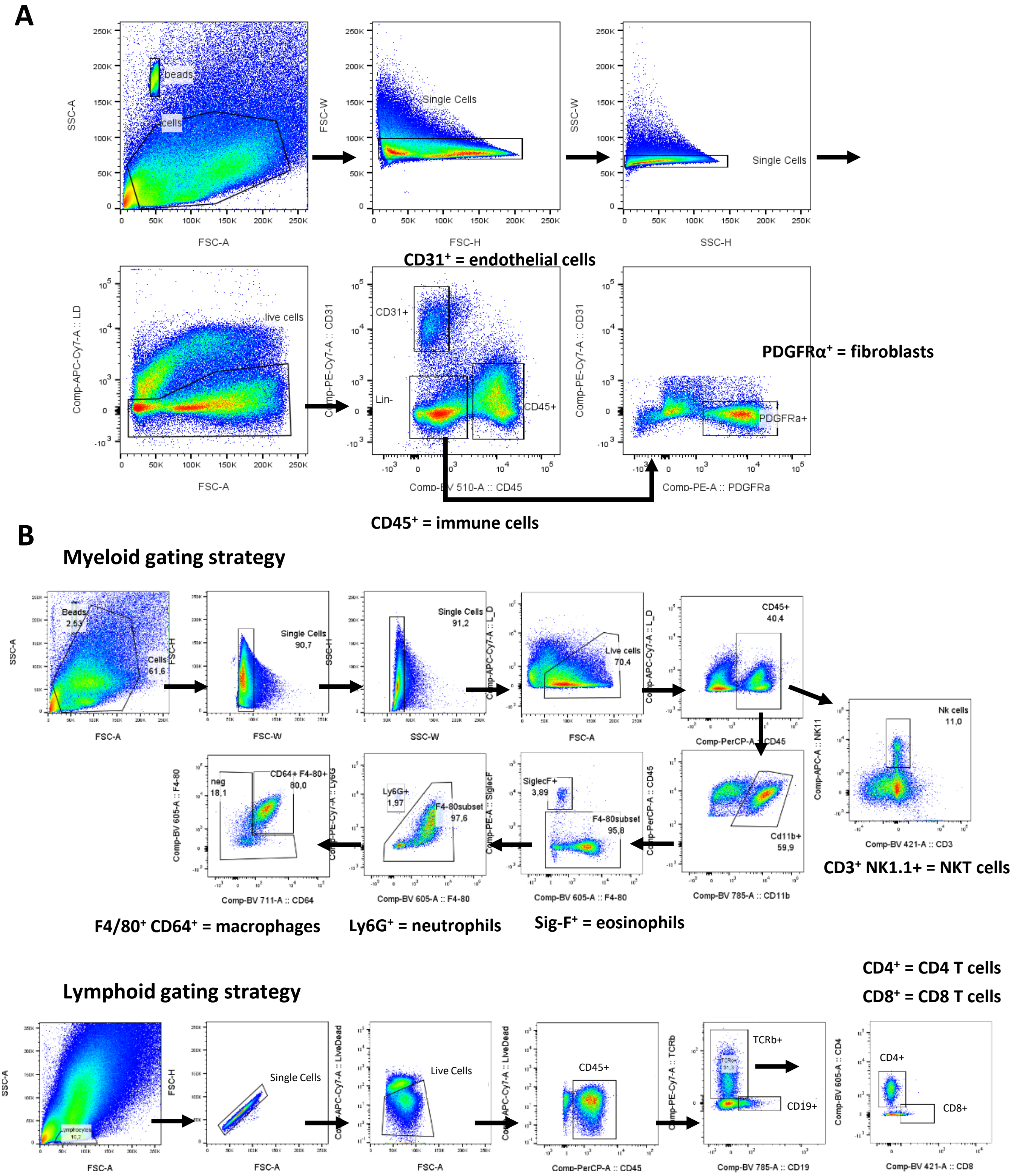

Supplementary Figure 5: Gating strategies to determine cellular composition of WAT in *Atg7*^{Ad} mice.

A) Gating strategy for CD45^{+}, CD31^{+}, and PDGFRα^{+} subsets within live cells.

B) Gating strategy for neutrophils (Ly6G^{+}), eosinophils (Siglec-F^{+}), macrophages (F4/80^{+} CD64^{+}), natural killer T (NKT) cells (CD3^{+} NK1.1^{+}), TCRβ^{+} CD8^{+} and CD4^{+} T cells within CD45^{+} live cells.

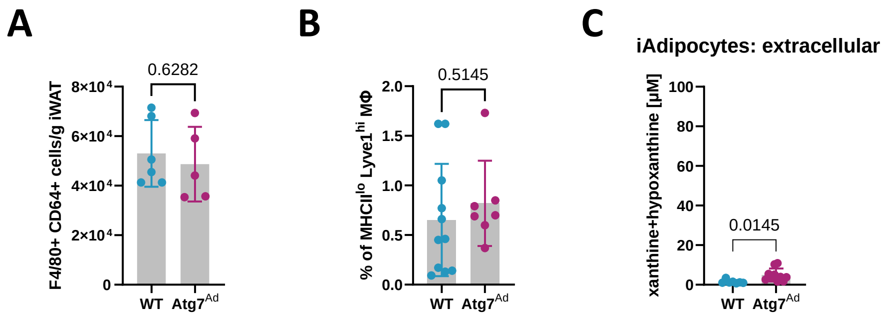

Supplementary Figure 6: *Atg7^{Ad}* iWAT lacks macrophage accumulation and shows modest but significantly increased xanthine and hypoxanthine secretion.

A) Flow cytometry analysis of F4/80^{+} CD64^{+} macrophage number in iWAT. n = 5 (*Atg7^{Ad}*) and 6 (WT) mice.

B) Quantification of perivascular macrophages identified as MHCII^{low} Lyve1^{high} by flow cytometry presented as frequency of total macrophages. n = 7 (*Atg7^{Ad}*) and 11 (WT) mice.

C) Concentration of xanthine and hypoxanthine secreted from iWAT adipocytes cultured over 24 hours *ex vivo*. Adipocytes were isolated from WT and *Atg7^{Ad}* mice fed with HFD for 16 weeks. n = 7 (WT) and 10 (*Atg7^{Ad}*) mice.

Data are presented as mean ± SD. Dots represent individual biological replicates. Data are representative (A) or merged from 3 independent experiments (B-C). Statistical analysis by unpaired t-test (A-C).

Supplementary Table 1: Materials and resources used in this study.

Antibodies	Source	Identifier
Rabbit anti-LC3 (1:1500)	Sigma-Aldrich	L8918
Rabbit anti-vinculin (1:2000)	Cell Signalling Technology	13901
Rabbit anti-caspase 3 (1:1000)	Cell Signalling Technology	9662
Rabbit IR Dye 800 CW (1:10000)	LI-COR	926-32211
BV510 anti-mouse CD45, clone 30-F11 (1:200)	BD Biosciences	563891
PE-Cyanine7 anti-mouse CD31, clone 390 (1:200)	Invitrogen	25-0311-81
PE anti-mouse CD140a (PDGFRa), clone APA5 (1:200)	Biolegend	135905
PerCP anti-mouse CD45, clone 30-F11 (1:200)	Biolegend	103130
BV785 anti-mouse/human CD11b, clone M1/70 (1:200)	Biolegend	101243
BV605 anti-mouse F4/80, clone BM8 (1:200)	Biolegend	123133
BV711 anti-mouse CD64, clone X54-5/7.1 (1:200)	Biolegend	139311
PE anti-mouse CD170 (Siglec-F), clone S17007L (1:200)	Biolegend	155505
APC anti-mouse NK-1.1, clone PK136 (1:200)	Biolegend	108709
PE-Cyanine7 anti-mouse Ly6G, clone 1A8 (1:200)	Biolegend	127618
PE-Cyanine7 anti-mouse TCR b chain, clone H57-597	Biolegend	109221
BV785 anti-mouse CD19, clone 6D5 (1:200)	Biolegend	115543
BV421 anti-mouse CD3ε, clone 145-2C11 (1:200)	Biolegend	100335
Pacific Blue anti-mouse CD8a, clone 53.6.-7 (1:400)	Biolegend	100725
BV605 anti-mouse CD4, clone GK1.5 or RM4-5 (1:400)	Biolegend	100451
PE anti-mouse CD45.1, clone A20 (1:200)	Biolegend	110707
BV711 anti-mouse CD45.2, clone 104 (1:200)	Biolegend	109847
APC anti-mouse CD9, clone MZ3 (1:200)	Biolegend	124811
PerCP/Cyanine5.5 anti-mouse CD63, clone NVG-2 (1:200)	Biolegend	143911
eFluor450 anti-mouse Lyve1, clone ALY7 (1:200)	Invitrogen	48-0443-82
PerCP/Cyanine5.5 anti-mouse I-A/I-E (MHCII), clone M5/114.15.2 (1:200)	Biolegend	107626
Mouse CD16/32 (1:200)	Biolegend	101302
Mouse anti-CD45 (1:500)	R&D Systems	AF114
Rabbit anti-CD68 (1:100)	Abcam	ab125212
Rat anti-F4/80 (1:100)	BioRad	MCA497A488T
Rabbit anti-Perilipin 1 (D1D8) (1:100)	Cell Signalling Technology	9349
Donkey Anti-Rat IgG H&L (Alexa Fluor 647) (1:500)	Abcam	ab150155
Donkey Anti-Rabbit IgG H&L (Alexa Fluor 555) (1:500)	Abcam	ab150074
Mouse FcR blocking reagent (1:200)	Miltenyi Biotec	130-092-575

Chemicals		
Tamoxifen	Sigma-Aldrich	T5648
DMEM high glucose medium	Sigma-Aldrich	D5796
RPMI-1640 medium	Sigma-Aldrich	R8758
RBC Lysis buffer	Invitrogen	00-4333-57
Phospate buffered saline (PBS)	Gibco	10010-023
Fatty acid-free BSA	Sigma-Aldrich	126609
HEPES	Gibco	15630-056
Liberase TL	Roche	5401020001
DNaseI	Roche	11284932001
EDTA	Invitrogen	15575-038
PBS 10X	VWR	437117K
TRI reagent	Sigma-Aldrich	T9424
cOmplete ULTRA tablets	Roche	5892791001
PhosSTOP	Roche	4906845001
BSA	Sigma-Aldrich	A7030
Skim milk powder	Sigma-Aldrich	70166
Prestained protein ladder	Abcam	ab116028
NuPAGE 4-12% Bis-Tris Gel	Invitrogen	NP0336BOX
NuPAGE MES SDS running buffer	Invitrogen	NP0002
NuPAGE Transfer buffer	Invitrogen	NP0006-1
Hypoxanthine	Sigma-Aldrich	H9636
Xanthine	Sigma-Aldrich	X7375
Adenosine	Sigma-Aldrich	F09333
Guanosine	Sigma-Aldrich	G6264
Forodesine hydrochloride	MedChemExpress	HY-16209
Q-VD-OPh	MedChemExpress	HY-12305

Citrate antigen retrieval solution (ARS1)	Vector Laboratories	H-3300
DAPI stock solution	ThermoFisher	D3571
Donkey serum	Sigma-Aldrich	D9663
Ethanol	Sigma-Aldrich	32221-2.5L
Glycerol	Sigma-Aldrich	G5516
Triton X-100	Sigma-Aldrich	T9284
Trizma base	Sigma-Aldrich	T6066
Xylene	Sigma-Aldrich	534056
Picro-sirius red solution	Abcam	ab24683
Haematoxylin Harris	Leica microsystems	3801560E
HCl 37%	VWR	20253.335
Eosin Y	Cell Path	RBC-0100-00A
Di-n-butylphthalate mounting media	Sigma-Aldrich	06522-100ml
Formalin solution, neutral buffered, 10%	Sigma-Aldrich	HT501128
Paraformaldehyde, 16% w/v aq. soln.	ThermoFisher	043368.9M
Calibrite 2-color beads	BD Biosciences	349502
D-(+)-Glucose	Sigma-Aldrich	G8270
Sucrose	Sigma-Aldrich	S9378
Methanol (Optima* LC/MS)	ThermoFisher	A456-1
Water	ThermoFisher	W5-1
Chloroform, Reagent ACS 99.8%, ACROS Organics	ThermoFisher	423550010
Chromacol™ GOLD-Grade Inert Glass Vials	ThermoFisher	13-622-351
9 mm autosampler vial screw thread caps (PTFE, silicone)	ThermoFisher	03-379-123
Sodium deoxycholate	Sigma-Aldrich	N/A
Dithiothreitol	Sigma-Aldrich	N/A
Chloroacetamide	Sigma-Aldrich	N/A
Lysyl Endopeptidase, mass spectrometry grade (LysC)	Wako	125-05061
Trypsin, sequence grade	Promega	VA9000
**Commercial assays**		
RNeasy Plus Micro Kit	Qiagen	74034
RNeasy Mini Kit	Qiagen	74106
High-capacity RNA-to-cDNA kit	Applied Biosystems	4387406
TaqMan Fast Advanced master mix	Applied Biosystems	4444557
LIVE/DEAD fixable near-IR dead cell stain kit	Invitrogen	L34976
LIVE/DEAD fixable aqua dead cell stain kit	Invitrogen	L34966
BCA assay	ThermoFisher	23227
IL1 beta mouse ELISA kit	Invitrogen	88-7013A-88
IL-10 mouse ELISA kit	Invitrogen	88-7105-86
IL-6 mouse ELISA kit	Invitrogen	88-7064-88
TGF beta-1 human/mouse ELISA kit	Invitrogen	88-8350-88
TNF alpha mouse ELISA kit	Invitrogen	88-7324-88
Adiponectin mouse ELISA kit	Invitrogen	KMP0041
Mouse Magnetic Luminex Assay (OPN)	Luminex	LXSAMSM
Leptin mouse ELISA kit	Invitrogen	KMC2281
Xanthine/Hypoxanthine Assay Kit	Abcam	ab155900
ATP Bioluminescence Assay Kit CLS II	Roche	11699695001
Amplex™ Red Xanthine/Xanthine Oxidase Assay Kit	Invitrogen	A22182
CD11b MicroBeads, human and mouse	Miltenyi Biotec	130-049-601
Alexa Fluor 488 antibody labeling kit	Invitrogen	A20181
**Deposited data**		
RNA-seq	This paper	GSE263837
proteomics	This paper	PXD052894
**Experimental models**		
Mouse: Adipoq-Cre	Charles River	25124
Mouse: Atg7$^{fl/fl}$	Komatsu et al., 2005	N/A
C57BL/6	bred in-house	N/A

**Oligonucleotides**		
Col1a1	ThermoFisher	Mm00801666_g1
Col3a1	ThermoFisher	Mm01254476_m1
Col6a3	ThermoFisher	Mm00711678_m1
Fn1	ThermoFisher	Mm01256744_m1
Mmp14	ThermoFisher	Mm00485054_m1
Timp1	ThermoFisher	Mm01341361_m1
Acta2	ThermoFisher	Mm00725412_s1
Il1b	ThermoFisher	Mm00434228_m1
Ppia	ThermoFisher	Mm02342430_g1
**Software and algorithms**		
R	R Project Team	https://www.r-project.org/
Prism 10	GraphPad	https://www.graphpad.com/
FlowJo v10.8.0	FlowJo	https://www.flowjo.com/
ImageJ	NIH	https://imagej.net/ij/
Zen 3.4	Zeiss	https://www.zeiss.com/
QuPath	Bankhead et al., 2017	https://qupath.github.io/
Excel	Microsoft	https://www.office.com/
Word	Microsoft	https://www.office.com/
PowerPoint	Microsoft	https://www.office.com/
Endnote	Endnote	https://endnote.com/
**Other**		
Rodent Diet with 60 kcal% Fat	ResearchDiets	D12492i
Rodent Diet with 10 kcal% Fat (Matching Sucrose to D12492)	ResearchDiets	D12450Ji
Glucose test strips	Abott	70815-71
Freedom Lite blood glucose monitoring system	Abott	ART20776-101
Microtainer tubes	BD Bioscience	365978
Microvette CB 300 LH	Sarstedt	16.443
pluriStrainer 300 um	pluriSelect	43-50300-01
Ceramic beads	Bertin Instruments	KT03961-1-003.2
MS columns	Miltenyi Biotec	130-042-201
LS columns	Miltenyi Biotec	130-042-401
24-well TPP tissue culture plate	Merck	Z707791
Nunc MaxiSorp ELISA uncoated plates	Biolegend	423501
Cell DIVE multiplex imager	GE	N/A
Axioscan 7 microscope slide scanner	Zeiss	N/A
Fortessa X-20 flow cytometer	BD Biosciences	N/A
NanoDrop	ThermoFisher	N/A
TissueLyser II	Qiagen	N/A
Precellys 24 homogenizer	Bertin Instruments	N/A
EZ2 Elite evaporator	Genevac	N/A
EASY-nLC 1200	ThermoFisher	N/A
Exploris 480	ThermoFisher	N/A
