# Supplementary material for: Autophagy acts as a brake on obesity-related fibrosis by controlling purine nucleoside signalling
Source: Nat Commun. 2025 Oct 17;16:9220. doi: 10.1038/s41467-025-64266-5 (PMC12534472; doi:10.1038/s41467-025-64266-5)
Supplement: Supplementary file 2 — Description of Additional Supplementary Files [file 41467_2025_64266_MOESM2_ESM.pdf]

Description of Additional Supplementary Files

File name: Supplementary Data 1
Description: Adipocyte metabolomics data.
Table contains the intensities (area under the curve or AUC) for all metabolites in the sample set (normalized to protein content), their abbreviated names (Name column) and KEGG IDs. Conditions are in a separate row, with three biological replicates per conditions denoted as Exp001 – 003. A one-way ANOVA statistical test has been applied (ANOVA column). Key to number of * in the Sig column: * $p<0.05$, ** $p<0.01$, *** $p<0.001$. Relative amounts for each metabolite are calculated using the calculated average amounts for each condition. The condition with the lowest average value (that is not 0) is set to 1 (the Norm_Av column).
