# Supplementary material for: Autophagy acts as a brake on obesity-related fibrosis by controlling purine nucleoside signalling
Source: Nat Commun. 2025 Oct 17;16:9220. doi: 10.1038/s41467-025-64266-5 (PMC12534472; doi:10.1038/s41467-025-64266-5)
Supplement: Supplementary file 4 — Reporting Summary [file 41467_2025_64266_MOESM4_ESM.pdf]

nature portfolio

Corresponding author(s): Anna Katharina Simon

Last updated by author(s): Aug 25, 2025

Reporting Summary

Nature Portfolio wishes to improve the reproducibility of the work that we publish. This form provides structure for consistency and transparency in reporting. For further information on Nature Portfolio policies, see our Editorial Policies and the Editorial Policy Checklist.

Statistics

For all statistical analyses, confirm that the following items are present in the figure legend, table legend, main text, or Methods section.

n/a	Confirmed	
☐	☒	The exact sample size (*n*) for each experimental group/condition, given as a discrete number and unit of measurement
☐	☒	A statement on whether measurements were taken from distinct samples or whether the same sample was measured repeatedly
☐	☒	The statistical test(s) used AND whether they are one- or two-sided<br>*Only common tests should be described solely by name; describe more complex techniques in the Methods section.*
☐	☒	A description of all covariates tested
☐	☒	A description of any assumptions or corrections, such as tests of normality and adjustment for multiple comparisons
☐	☒	A full description of the statistical parameters including central tendency (e.g. means) or other basic estimates (e.g. regression coefficient) AND variation (e.g. standard deviation) or associated estimates of uncertainty (e.g. confidence intervals)
☐	☒	For null hypothesis testing, the test statistic (e.g. *F*, *t*, *r*) with confidence intervals, effect sizes, degrees of freedom and *P* value noted<br>*Give P values as exact values whenever suitable.*
☒	☐	For Bayesian analysis, information on the choice of priors and Markov chain Monte Carlo settings
☒	☐	For hierarchical and complex designs, identification of the appropriate level for tests and full reporting of outcomes
☐	☒	Estimates of effect sizes (e.g. Cohen's *d*, Pearson's *r*), indicating how they were calculated

Our web collection on statistics for biologists contains articles on many of the points above.

Software and code

Policy information about availability of computer code

Data collection	Transcriptomics data quality control was performed using a pipeline readqc.py (https://github.com/cgat-developers/cgat-flow) and paired-end reads were aligned to GRCm38/Mm10 reference genome using the pseudoalignment method Salmon (Patro et al., 2017) and Kallisto (Bray et al., 2016).<br>Proteomics data were colected using Exploris 480 in data-independent acquisition (DIA) mode.<br>Metabolomics data were quantified with Maven (v 8.1.27.11).<br>Immunohistochemistry images were collected with Zeiss ZEN (blue edition, version 3.4).<br>Immunofluorescence images were collected with IN Cell Analyzer 2500 software and Zeiss ZEN (blue edition, version 3.4).<br>Real-time PCR data were collected with QuantStudio (version 1.4).<br>Flow cytometry data were collected with BD FACSDiva v8.01.
Data analysis	Transcriptomics data analysis was performed with DEseq2 (v1.38.3), limma package (v3.54.1), and clusterProfiler package (v4.6.0) in R.<br>Proteomics data analysis was performed with DIA-NN version 1.8.1 and limma package (v3.54.1).<br>Single nucleus RNAseq data analysis was performed with Seurat package for single-cell RNA-seq analysis.<br>Image analysis was performed with Zeiss ZEN (blue edition, version 3.4), QuPath (version 0.4.2) and Image J (v1.53t).<br>Flow cytometry analysis was performed with Flow Jo v10.8.0.<br>Statistical analysis was performed with Prism v10.<br>Western blot analysis was performed with Image Studio Lite v5.2.

For manuscripts utilizing custom algorithms or software that are central to the research but not yet described in published literature, software must be made available to editors and reviewers. We strongly encourage code deposition in a community repository (e.g. GitHub). See the Nature Portfolio guidelines for submitting code & software for further information.

Data

Policy information about availability of data

All manuscripts must include a data availability statement. This statement should provide the following information, where applicable:

- Accession codes, unique identifiers, or web links for publicly available datasets
- A description of any restrictions on data availability
- For clinical datasets or third party data, please ensure that the statement adheres to our policy

The authors declare that all the data supporting the findings of this study are available within the paper and its supplementary information and will be fully deposited into online databases shortly. RNA sequencing data reported in this paper are available at accession number GEO: GSE263837 and GSE306424. Proteomics data are available at ProteomeXchange ID: PXD052894. Metabolomics data are available as Supplementary Data.

Research involving human participants, their data, or biological material

Policy information about studies with human participants or human data. See also policy information about sex, gender (identity/presentation), and sexual orientation and race, ethnicity and racism.

Reporting on sex and gender	N/A
Reporting on race, ethnicity, or other socially relevant groupings	N/A
Population characteristics	N/A
Recruitment	N/A
Ethics oversight	N/A

Note that full information on the approval of the study protocol must also be provided in the manuscript.

Field-specific reporting

Please select the one below that is the best fit for your research. If you are not sure, read the appropriate sections before making your selection.

☒ Life sciences ☐ Behavioural & social sciences ☐ Ecological, evolutionary & environmental sciences

For a reference copy of the document with all sections, see nature.com/documents/nr-reporting-summary-flat.pdf

Life sciences study design

All studies must disclose on these points even when the disclosure is negative.

Sample size	Sample size is listed under each figure. Sample sizes were not pre-determined using statistical methods and are similar to previously reported publications (PMID: 36795015). Where required, effect sizes were estimated via pilot experiments. Normal distribution of data was tested before applying parametric or nonparametric testing.
Data exclusions	No data were excluded for the analysis.
Replication	All in vivo experiments used biological replicates and in vitro experiments additionally used technical replicates. Each experiment was conducted at least as two independent replicates. All experiments included in the manuscript were reproducible and representative data are shown in the figures.
Randomization	Mice were sex and age-matched to generate experimental groups. Experimental cages were balanced for genotypes to minimise cage effects. Littermate controls were used where possible. In vitro samples were allocated randomly between control and treatment conditions. Sample acquisition was performed randomly.
Blinding	While experimental groups were assembled in a non-blinded manner to ensure equal proportions of both wild type and knockout mice, mouse genotypes within individual cages were blinded during data collection. Analysis of images and assay data was also blinded.

Reporting for specific materials, systems and methods

We require information from authors about some types of materials, experimental systems and methods used in many studies. Here, indicate whether each material, system or method listed is relevant to your study. If you are not sure if a list item applies to your research, read the appropriate section before selecting a response.

Materials & experimental systems

n/a	Involved in the study
	☒ Antibodies
☒	Eukaryotic cell lines
☒	Palaeontology and archaeology
	☒ Animals and other organisms
☒	Clinical data
☒	Dual use research of concern
☒	Plants

Methods

n/a	Involved in the study
☒	ChIP-seq
	☒ Flow cytometry
☒	MRI-based neuroimaging

Antibodies

Antibodies used

Rabbit anti-LC3 (1:1500) Sigma-Aldrich L8918
Rabbit anti-vinculin (1:2000) Cell Signalling Technology 13901
Rabbit anti-caspase 3 (1:1000) Cell Signalling Technology 9662
Rabbit IR Dye 800 CW (1:10000) LI-COR 926-32211
BV510 anti-mouse CD45, clone 30-F11 (1:200) BD Biosciences 563891
PE-Cyanine7 anti-mouse CD31, clone 390 (1:200) Invitrogen 25-0311-81
PE anti-mouse CD140a (PDGFRa), clone APA5 (1:200) Biolegend 135905
PerCP anti-mouse CD45, clone 30-F11 (1:200) Biolegend 103130
BV785 anti-mouse/human CD11b, clone M1/70 (1:200) Biolegend 101243
BV605 anti-mouse F4/80, clone BM8 (1:200) Biolegend 123133
BV711 anti-mouse CD64, clone X54-5/7.1 (1:200) Biolegend 139311
PE anti-mouse CD170 (Siglec-F), clone S17007L (1:200) Biolegend 155505
APC anti-mouse NK-1.1, clone PK136 (1:200) Biolegend 108709
PE-Cyanine7 anti-mouse Ly6G, clone 1A8 (1:200) Biolegend 127618
PE-Cyanine7 anti-mouse TCR b chain, clone H57-597 Biolegend 109221
BV785 anti-mouse CD19, clone 6D5 (1:200) Biolegend 115543
BV421 anti-mouse CD3ε, clone 145-2C11 (1:200) Biolegend 100335
Pacific Blue anti-mouse CD8a, clone 53.6.-7 (1:400) Biolegend 100725
BV605 anti-mouse CD4, clone GK1.5 or RM4-5 (1:400) Biolegend 100451
PE anti-mouse CD45.1, clone A20 (1:200) Biolegend 110707
BV711 anti-mouse CD45.2, clone 104 (1:200) Biolegend 109847
APC anti-mouse CD9, clone MZ3 (1:200) Biolegend 124811
PerCP/Cyanine5.5 anti-mouse CD63, clone NVG-2 (1:200) Biolegend 143911
eFluor450 anti-mouse Lyve1, clone ALY7 (1:200) Invitrogen 48-0443-82
PerCP/Cyanine5.5 anti-mouse I-A/I-E (MHCII), clone M5/114.15.2 (1:200) Biolegend 107626
Mouse CD16/32 (1:200) Biolegend 101302
Mouse anti-CD45 (1:500) R&D Systems AF114
Rabbit anti-CD68 (1:100) Abcam ab125212
Rat anti-F4/80 (1:100) BioRad MCA497A488T
Rabbit anti-Perilipin 1 (D1D8) (1:100) Cell Signalling Technology 9349
Donkey Anti-Rat IgG H&L (Alexa Fluor 647) (1:500) Abcam ab150155
Donkey Anti-Rabbit IgG H&L (Alexa Fluor 555) (1:500) Abcam ab150074
Mouse FcR blocking reagent (1:200) Miltenyi Biotec 130-092-575

Validation

All antibodies come from commercial vendors, which independently validated antibodies (Biolegend, BD Biosciences, R&D Systems, Sigma-Aldrich, Cell Signalling Technology, Invitrogen, BioRad, Abcam, Miltenyi Biotec). Antibodies were used in applications where previously validated. Where noted in the figures, we performed further validation using relevant isotype controls. Detailed information about antibody validation can be obtained from the manufacturer's website using the catalog numbers listed above.

Animals and other research organisms

Policy information about studies involving animals; ARRIVE guidelines recommended for reporting animal research, and Sex and Gender in Research

Laboratory animals

Adipoq-CreERT2 mice (Sassmann et al., 2010) were purchased from Charles River, UK (JAX stock number: 025124) and crossed to Atg7fl/fl mice (Komatsu et al., 2005). Wild-type C57BL/6J or B6.SJL.CD45.1 mice were bred in-house.

Wild animals

The study did not involve wild animals.

Reporting on sex

Our findings apply to both male and female mice, and we did not observe any gender effects throughout our study. Due to this our study design did not restrict the use of a particular sex.

Field-collected samples

The study did not involve samples collected from the field.

Ethics oversight

Mice were bred and maintained under specific pathogen-free conditions at the Kennedy Institute of Rheumatology, University of

Ethics oversight | Oxford. All experiments were performed in accordance to approved procedures by the Local Review Committee and the Home Office under the project license (PPL30/3388 and P01275425).

Note that full information on the approval of the study protocol must also be provided in the manuscript.

Plants

Seed stocks: N/A

Novel plant genotypes: N/A

Authentication: N/A

Flow Cytometry

Plots

Confirm that:

☒ The axis labels state the marker and fluorochrome used (e.g. CD4-FITC).

☒ The axis scales are clearly visible. Include numbers along axes only for bottom left plot of group (a 'group' is an analysis of identical markers).

☒ All plots are contour plots with outliers or pseudocolor plots.

☒ A numerical value for number of cells or percentage (with statistics) is provided.

Methodology

Sample preparation: Adipose tissue digestion was performed in DMEM containing 1 % fatty acid-free BSA, 5 % HEPES, 0.2 mg/ml Liberase TL (Roche), and 20 µg/mL DNaseI. Tissues were minced and incubated for 25-30 min at 37°C at 180 rpm. Digested tissue was strained through a 300 µm mesh and the digestion was quenched by addition of DMEM with 2 mM EDTA. Adipocyte and stromal vascular fraction were separated by 7 min centrifugation at 500 g. For surface staining, stromal vascular fraction cells were incubated with fluorochrome-conjugated antibodies, LIVE/DEAD Fixable Stains and Fc receptor block antibody for 20 min at 4°C. This was followed by a 10 min fixation with 4% PFA at room temperature.

Instrument: Fortessa X-20 and Aria (BD Biosciences)

Software: Data were analysed with FlowJo v10.8.0.

Cell population abundance: Abundances of individual cell populations are provided in the figures.
The purity of macrophages sorted for RNA sequencing was above 90%. Briefly, stromal vascular fraction was incubated with viability dye, anti-CD16/32 (FcBlock) and surface flow antibodies and sorted by BD Aria FACS.
To isolate macrophages for primary cell culture, stromal vascular fraction was enriched for CD11b+ cells with CD11b MicroBeads (Miltenyi Biotec) according to manufacturers' instructions after red blood cell lysis. Purity validated by flow cytometry was >80%.

Gating strategy: Cells were gated based on FSC/SSC properties followed by exclusion of doublets. Viable cells were gated by excluding Live/Dead+ cells. Further downstream gating strategy is indicated in the supplementary information.

☒ Tick this box to confirm that a figure exemplifying the gating strategy is provided in the Supplementary Information.
